# Droplet Fusion in Oil-in-Water Pickering Emulsions

**DOI:** 10.3389/fchem.2018.00213

**Published:** 2018-06-12

**Authors:** Catherine P. Whitby, Floriane Bahuon

**Affiliations:** Institute of Fundamental Sciences, Massey University, Palmerston North, New Zealand

**Keywords:** particle-stabilized emulsion, limited coalescence, compound drop, Janus droplet, multiple emulsion

## Abstract

We have formed compound droplets made of two or more drops of immiscible oils by temporarily destabilizing Pickering oil-in-water emulsions. The emulsions used are synergistically stabilized by mixtures of cationic surfactant and negatively-charged particles. They are highly sensitive to the concentration of surfactant present in the emulsions. We took advantage of transient droplet coalescence events that are triggered by reducing the surfactant concentration to fuse together drops of immiscible oils. This study provides guidelines for designing compound droplets by transient (or limited) coalescence in Pickering emulsions. We show that the possible geometries of particle-stabilized compound drops are determined by the interfacial tensions and relative volumes of the drops fused together. The implications of our results for designing strategies to fabricate multiphase drops are discussed.

## Introduction

Compound droplets consist of drops of two (or more) immiscible fluids that have been fused together (Johnson and Sadhal, 1985; Neeson et al., 2012). Emulsions containing drops with multiple compartments have the potential to protect incompatible ingredients in foods (Muschiolik, 2007), pharmaceutics (Zhao, 2013), and cosmetics (Tadros, 1992). For example, multiple emulsions (Silva et al., 2016) have long been considered for use as vehicles for encapsulating sensitive ingredients in emulsion based food products (Pawlik et al., 2010; Sapei et al., 2012; McClements, 2015; Muschiolik and Dickinson, 2017). Yet the controlled production of large volumes of complex emulsions remains challenging. Advances in microfluidic emulsification techniques led to breakthroughs in the precise fabrication of small volumes of multiple emulsion droplets (Nisisako et al., 2005). They also enabled the assembly of compound droplets with morphologies that were previously unattainable. Novel configurations, such as Janus droplets, can form under the extreme flow conditions achieved in microfluidic channels (Choi et al., 2013). These developments have rejuvenated interest in designing robust and convenient routes for fusing drops of immiscible liquids together on a scale larger than that achieved in microfluidic channels (Hasinovic and Friberg, 2011; Fryd and Mason, 2014; Weyer et al., 2015; Ge et al., 2017; Wei et al., 2017). In this brief report, we describe a new approach that uses nanoparticles to control the fabrication of drops with multiple compartments.

Particles present during emulsion formation assemble at oil–water interfaces (of interfacial tension, γ) by becoming partially immersed in both liquids and forming a three phase oil-water-particle contact angle, θ. This is energetically favorable because attached particles reduce the total interfacial area between the oil and water. For spherical particles (of radius *r*_p_) with θ < 90°, the free energy of attaching a particle to a drop (Levine et al., 1989; Binks and Lumsdon, 2000) is given by $\Delta G=\pi \gamma r_{p}^{2}{\left(1-cos\theta \right)}^{2}$. If the total number of particles present during emulsion formation is not sufficient to fully coat the oil–water interface, the drops coalesce together until a critical degree of surface coverage by the particles is reached (Arditty et al., 2003). Surfactants are often mixed with particles to optimize the particle wettability at the oil–water interface (Binks et al., 2007; Eskandar et al., 2007; Wang et al., 2010; Reger et al., 2011; Hu et al., 2015; Wang and Wang, 2016). Synergistic stabilization of emulsions by mixtures of surfactant and particles makes them highly sensitive to changes in the concentration of surfactant present in the continuous phase (Binks and Whitby, 2005). We took advantage of the disruption to the interfacial particle layer caused by reducing the surfactant concentration to trigger fusion of droplets of immiscible oil in particle-stabilized (or Pickering) emulsions.

## Materials and methods

### Emulsion formation and fusion

Oil-in-water emulsions were stabilized by mixtures of hydrophilic fumed silica nanoparticles (Wacker Chemie N20) and hexadecyltrimethyl ammonium bromide (CTAB, 99% purity, Sigma Aldrich). The silica nanoparticles were obtained as a powder, with an average primary particle size of 10 nm. The powder dispersed in water as aggregates with an average size of 200 nm. The aggregates have a zeta potential of −37 ± 4 mV (measured using a Malvern Zetasizer). For experiments involving drops of mutually immiscible oils we used olive oil (highly refined and of low acidity) and 5 cSt silicone oil. Both oils were obtained from Sigma Aldrich and passed through a column of chromatographic alumina prior to use.

To optimize particle wettability and hence emulsion stability, the silica nanoparticles were dispersed in aqueous solutions of 0.1 mM CTAB by sonication in an ultrasound bath (Soniclean 160 T, 70 W power, ~44 kHz operating frequency) for 1 h. At these surfactant concentrations, the silica particles flocculated and the dispersions sedimented over time when left to stand. The pH of the dispersions was about 5.8.

Emulsions of each type of oil (at drop volume fractions 0.1 ≤ ϕ ≤ 0.4 and a total volume of 15 mL) were prepared by homogenizing appropriate volumes of the oil with the aqueous silica dispersions (0.5 wt.% in 0.1 mM CTAB) using a PowerGen 125 mechanical mixer with a 7 mm diameter shaft operated at 30,000 rpm for 2 min. The drops in the oil-in-water emulsions remain dispersed and do not coalesce for a few months under quiescent conditions. The emulsions release water over time due to the oil drops creaming, however there was no evidence of an excess of silica particles in the water, indicating that all the particles present in the emulsions were located at the droplet surfaces.

Destabilization was triggered by lowering the surfactant concentration in the emulsions. The emulsions were gently diluted in sufficient water to lower the CTAB concentration to ≤ 0.01 mM. To create multi-compartment emulsion droplets, samples of the emulsions of each type of oil were gently mixed by hand together with sufficient water to lower the CTAB concentration to 0.01 mM.

### Emulsion characterization

To visualize the emulsion microstructure by optical microscopy we used a Zeiss Axiophot Microscope. To visualize the fused droplets by confocal microscopy we used a Leica SP5 DM6000B Scanning Confocal Microscope. Prior to emulsion formation the particles were stained with Nile Blue (0.1 mM in water). Nile Red (0.1 mM in oil) was used for staining the oil. Preliminary studies confirmed that the presence of the dyes did not alter emulsion structure or stability. The Nile Blue was excited at 633 nm and the Nile Red at 496 nm. Fluorescence intensity data for Nile Blue and Nile Red were collected in two separate channels corresponding to 520–570 and 653–750 nm, respectively. Each line of pixels in an image was scanned sequentially for Nile Red and Nile Blue fluorescence to avoid interference due to cross-fluorescence.

The drop size distributions in the emulsions were measured by static light scattering using Mastersizer 3000. The average of the volume-weighted mean diameter of three distributions measured on different samples of the same emulsion was calculated. The typical standard deviation of the mean drop diameters was 15 μm.

A glass cover slip was used as a planar substrate for wetting measurements. The glass cover slips were immersed in oil (olive oil or silicone oil) contained in a rectangular cell. A drop of surfactant solution was then placed onto the glass surface and left to equilibrate for 30 min. The advancing contact angle was measured through aqueous phase using a KSV CAM 200. The average of contact angle measurements on five drops was calculated. The typical standard deviation of the contact angle measurements was 8°.

Interfacial tensions were measured using an Attensiometer digital tensiometer with a platinum du Nouy ring. The average of three measurements of the interfacial tension was calculated. The typical standard deviation of the interfacial tension measurements was 3 mN m^−1^.

## Results and discussion

We studied fusion in oil-in-water emulsions stabilized by mixtures of negatively charged silica nanoparticles and a cationic surfactant, hexadecyltrimethyl ammonium bromide. It should be noted that it was not possible to stabilize emulsions in the presence of the silica nanoparticles alone (the three phase oil-water-particle contact angle, θ, of silica particles is ~0°). In the absence of particles, emulsions prepared from solutions of CTAB at the concentrations studied are also unstable to phase separation. Preliminary experiments established the criteria for forming dilute oil-in-water emulsions of each type of oil that were stable to coalescence. Emulsions of a single type of oil were typically stabilized by mixtures of 0.5 wt.% particles and CTAB at concentrations >0.01 mM. At this particle concentration there was no evidence of excess (unattached) particles in the emulsions. The emulsions were studied at drop volume fractions, ϕ, ranging from 0.1 to 0.4, since the drops in more concentrated emulsions were unstable to coalescence. This is consistent with previous findings (Binks and Whitby, 2005) that adsorption of CTAB on silica nanoparticles alters the particle wettability and stability to flocculation and hence improves emulsion stability to coalescence.

Destabilization was triggered by lowering the surfactant concentration in the emulsions to ≤ 0.01 mM. Diluting emulsions of a single type of oil in pure water to reduce the surfactant concentration caused the drops to undergo coalescence to a limited extent. For example, the average drop size measured by dynamic light scattering in emulsions (at a drop volume fraction of ϕ = 0.01 after dilution) increased by 10% within 2 days of reducing the surfactant concentration. Further changes in the drop size distributions were not observed in very dilute emulsions. Larger increases in the average drop size were observed in emulsions at higher drop volume fractions. The average drop size in the emulsions at drop volume fractions, ϕ = 0.04 increased by up to 50% within 2 days of reducing the surfactant concentration. A layer of oil was observed on top of the emulsions (at ϕ = 0.04) after 7 days of standing at rest. A layer of emulsion did remain, however, indicating that destabilization was only temporary, as a fraction of the drop population had stopped undergoing coalescence.

The coalescence stability of Pickering emulsions is determined by the lifetime of the particle-stabilized thin films formed between drops that come into close contact. We propose that reducing the surfactant concentration in the emulsions alters the particle wettability and hence the position of the attached particles at the drop surfaces. This was tested by measuring the contact angle of aqueous surfactant solutions on planar glass surfaces (microscope slides) under oil. Under silicone oil, θ decreased from about 93° to 55° as the CTAB concentration was reduced from 0.1 to 0.01 mM. The contact angle under olive oil decreased from 108° to 67° over the same surfactant concentration range. The contact angle of the glass surfaces was about 90° at high surfactant concentrations due to the surfactant molecules adsorbing in conformations where the cationic head-group neutralizes an anionic site on the surface and the hydrocarbon tail is exposed, making the surface hydrophobic. This wettability is optimal for attaching particles to drop surfaces. Reducing the surfactant concentration caused desorption of surfactant molecules from the glass surface, reducing its hydrophobicity. A reduction in the wettability of the silica nanoparticles at the droplet surfaces would reduce the energy required to detach the particles from the drop surfaces, increasing the probability of thin film rupture and coalescence between drops that come into close contact. The disruption to the interfacial particle layer causes the drops to coalesce together until the density of the particles coating the surfaces of the merging drops is sufficient to inhibit further instability.

**Figure 1:**
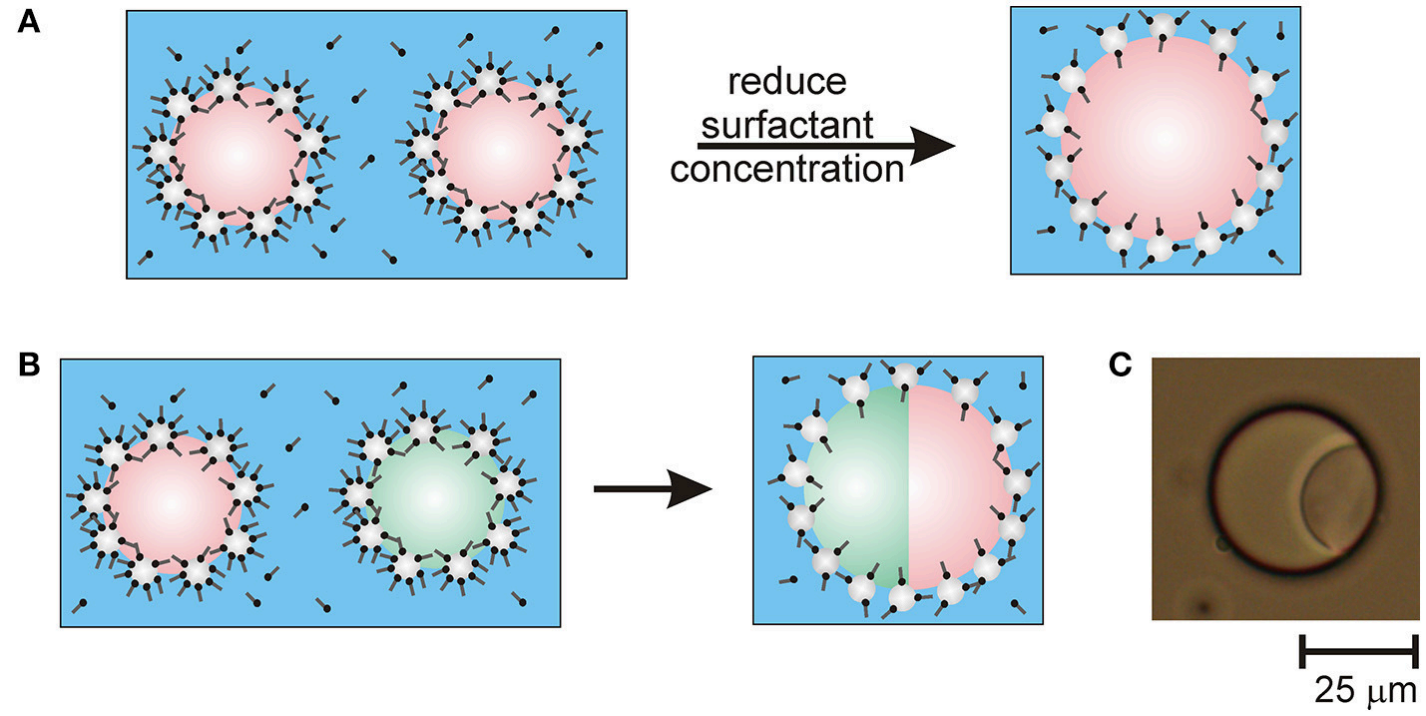
Schematics of Pickering emulsion destabilization caused by reducing the surfactant concentration in emulsions stabilized by mixtures of particles and surfactant. **(A)** Destabilizing an emulsion containing drops of a single type of oil in water causes the drops to undergo limited coalescence. **(B)** Destabilizing an emulsion containing drops of two different immiscible types of oil causes the drops to fuse with multiple together into drops compartments. **(C)** Optical microscope image of a fused droplet containing compartments of olive oil and silicone oil.

The proposed mechanism for the limited coalescence caused by reducing the surfactant concentration in the Pickering emulsions is illustrated in the schematic in Figure 1A. In the schematic, the orientation of the surfactant molecules adsorbed on particle surfaces in contact with oil is depicted as the same as the orientation of the surfactant molecules on particle surfaces in contact with water. This is because the mechanism by which emulsions prepared in the presence of mixtures of particles and surfactants are stabilized remains unclear. Although it is hypothesized that it is due to unique flocculation mechanisms between particles and surfactant, little is known about how particles and surfactant arrange at drop surfaces (Maestro et al., 2015). It is not known whether the orientation of the surfactant molecules changes on the part of the particle surfaces that become immersed in the oil.

It should be noted that lowering the surfactant concentration below 0.01 mM CTAB tended to cause uncontrolled coalescence. This led to phase separation of the emulsions during mixing (dilution). At very low surfactant concentrations there are insufficient surfactant molecules available to adsorb to the particle surfaces and increase the particle hydrophobicity for re-stabilization of the emulsion to occur. Thus, there is a range of surfactant concentrations over which coalescence (and fusion, as shown shortly) is limited and ceases after some time.

**Figure 2:**
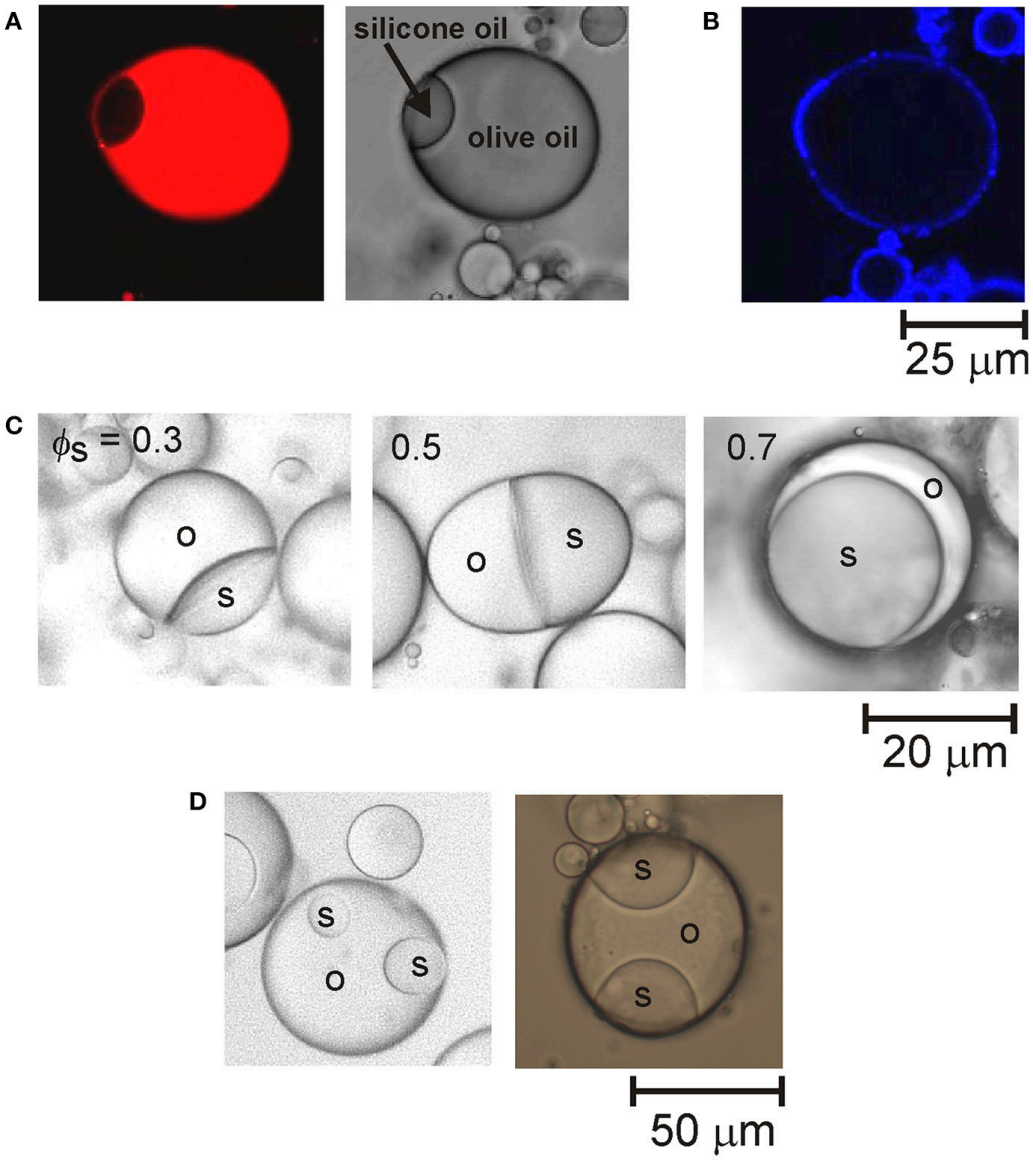
**(A)** Confocal fluorescence image (left) and an optical microscope image (right) of a fused droplet of olive oil and silicone oil. The confocal image shows the fluorescence from the Nile Red staining the olive oil compartment. **(B)** Confocal fluorescence image of the particle shell encapsulating the fused droplet shown in **(A)**. **(C)** Optical microscope images of fused droplets of olive oil (o) and silicone oil (s) at different volume fractions of silicone oil (ϕ_s_) in the compound drops. The curvature of the interface between the two oil compartments changes from concave to planar to convex as the volume fraction of the silicone oil compartment increases. **(D)** Optical microscope images of fused droplets containing more than two compartments.

To create multi-compartment emulsion droplets, samples of the emulsions of each type of oil were gently mixed by hand together with sufficient water to lower the CTAB concentration to 0.01 mM. A schematic of the droplet fusion process expected to occur is shown in Figure 1B. After allowing the mixed emulsions to rest for 1 day, they were examined by optical microscopy. Figure 1C shows an example of the anisotropic droplet microstructures observed. The compound drops are composed of droplets of olive oil and silicone oil that have fused together due to the transient destabilization of the particle layers coating the drops caused by reducing the CTAB concentration. Since the oils used are not miscible, the drops fuse rather than completely merging their contents. This was confirmed by staining the oil phases in the emulsions with fluorescent dyes. To differentiate between the olive oil and PDMS compartments, the olive oil was stained with Nile Red or pyrene prior to emulsion formation. For example, Figure 2A shows fluorescence from Nile Red staining the olive oil compartment of the compound drop. Nile Red is a red phenoxazone dye that dissolves readily in olive oil (which is mainly composed of triglycerides) rather than polydimethylsiloxane (PDMS, or silicone oil).

The compound droplet microstructures are stabilized by layers of particles coating the perimeters of the fused drops. This was confirmed by staining the particles with Nile Blue prior to emulsion formation. Figure 2B shows that staining the particles rendered the external surfaces of the droplets visible. The internal interfaces between the droplet compartments are not coated by particles. This means that particles are displaced from the olive oil–silicone oil interface that forms as the thin films between merging droplets rupture during droplet fusion. The displacement of the particles from the oil–oil interfaces can be accounted for by considering the interfacial tensions of the compound droplets. Interfacial tensions of γ_ow_ = 16.4 mN m^−1^ and γ_sw_ = 38.5 mN m^−1^ were measured for the olive oil–water and PDMS–water interfaces, respectively. The interfacial tension of the olive oil–PDMS interface was estimated to be much lower (γ_os_ = 1 mN m^−1^). The lower interfacial energy trapping the particles at the oil–oil interfaces means that the particles tend to diffuse away from the area of contact between the droplets and increase the density of particles at the oil–water interfaces of fused drops.

The majority of fused drops formed in these experiments had only two compartments. They had partially engulfed, or Janus topologies, rather than completely engulfed configurations. This means that the two drops (or compartments) of immiscible oil have a common interface. The remaining portion of both drop surfaces are exposed to the water. A key factor that influences the shape of the two compartments is the relative volumes of the drops. Figure 2C shows examples of the morphology of Janus droplets formed by fusing drops of silicone oil and olive oil of different sizes. The curvature of the oil–oil interfaces changes as the volume fraction of the silicone oil compartment increases. The droplet configuration can be rationalized by considering the three interfacial tensions involved. If we consider a drop of olive oil placed at a silicone oil–water interface, the spreading coefficient is given by *S* = γ_*sw*_ − (γ_*ow*_ + γ_*os*_). The coefficient must be a positive number for spreading to occur spontaneously. Due to the very low interfacial tension between the two oils, the spreading coefficient effectively depends on the difference between the olive oil–water and silicone oil–water interfacial tensions. The higher interfacial tension between silicone oil and water means that the olive oil will spontaneously spread over the silicone oil. Thus, fused droplets containing a relatively small compartment of silicone oil adopt a configuration where the silicone oil forms a small lens pinned on the surface of the olive oil compartment. Fused drops containing a small compartment of olive oil, on the other hand, adopt a configuration where the olive oil spreads around the surface of the silicone oil droplet. Thus, the transition between the different morphologies is consistent with theoretical predictions of the spreading behavior (Torza and Mason, 1969) of the two oils studied.

A small proportion of drops with several compartments was observed after mixing together emulsions containing relatively large oil volume fractions. This indicates that the fusion of three or four droplets is also possible during the transient destabilization. The more complex multiphase droplet structures that form consist of a number of small droplets of one type of oil located around the edge of a larger, central droplet of the other type of oil, as shown in Figure 2D.

The focus of this work was on demonstrating the formation of compound drops by using a well-known combination of particles and surfactant as synergistic stabilizers. CTAB is commonly used to modify the wettability of nanoparticles, however it is a toxic surfactant. A key step in the development of particle-stabilized compound drops for use in therapeutic or food formulations will involve using acceptable ingredients as stabilizers. The wide range of combinations of surfactants and particles used to synergistically stabilize emulsions (Dickinson, 2010) should make this a versatile and general route for preparing (kinetically) stable multi-compartment drops.

Compound drops stabilized by nanoparticles may have potential applications for the controlled release of sensitive ingredients. The formation of nanoparticle layers at fluid interfaces has been shown to be a highly effective strategy for protecting sensitive compounds encapsulated within simple oil drops and controlling their release (Eskandar et al., 2009; Kargar et al., 2011). Further experiments are required to investigate the encapsulation efficiency of particle-coated compound droplets.

## Conclusion

In conclusion we have established a straightforward process for generating compound drops from two or more immiscible oils with minimal agitation. Using the limited coalescence behavior of Pickering emulsions to fuse drops together opens up the possibility of creating large numbers of drops with multiphase structures. Applications of compound drops may benefit from the enhanced stability of particle-stabilized drops.

## Author contributions

CW guided the design of experiments. FB conducted the experiments. CW and FB analyzed the data and wrote the manuscript.

### Conflict of interest statement

The authors declare that the research was conducted in the absence of any commercial or financial relationships that could be construed as a potential conflict of interest.
